# Retrospective analysis and time series forecasting with automated machine learning of ascariasis, enterobiasis and cystic echinococcosis in Romania

**DOI:** 10.1371/journal.pntd.0009831

**Published:** 2021-11-01

**Authors:** Johannes Benecke, Cornelius Benecke, Marius Ciutan, Mihnea Dosius, Cristian Vladescu, Victor Olsavszky

**Affiliations:** 1 Department of Dermatology, Venereology and Allergology, University Medical Center and Medical Faculty Mannheim, University of Heidelberg, and Center of Excellence in Dermatology, Mannheim, Germany; 2 Barcelona Institute for Global Health, University of Barcelona, Barcelona, Spain; 3 National School of Public Health Management and Professional Development, Bucharest, Romania; 4 University Titu Maiorescu, Faculty of Medicine, Bucharest, Romania; Universite de Montreal, CANADA

## Abstract

The epidemiology of neglected tropical diseases (NTD) is persistently underprioritized, despite NTD being widespread among the poorest populations and in the least developed countries on earth. This situation necessitates thorough and efficient public health intervention. Romania is at the brink of becoming a developed country. However, this South-Eastern European country appears to be a region that is susceptible to an underestimated burden of parasitic diseases despite recent public health reforms. Moreover, there is an evident lack of new epidemiologic data on NTD after Romania’s accession to the European Union (EU) in 2007. Using the national ICD-10 dataset for hospitalized patients in Romania, we generated time series datasets for 2008–2018. The objective was to gain deep understanding of the epidemiological distribution of three selected and highly endemic parasitic diseases, namely, ascariasis, enterobiasis and cystic echinococcosis (CE), during this period and forecast their courses for the ensuing two years. Through descriptive and inferential analysis, we observed a decline in case numbers for all three NTD. Several distributional particularities at regional level emerged. Furthermore, we performed predictions using a novel automated time series (AutoTS) machine learning tool and could interestingly show a stable course for these parasitic NTD. Such predictions can help public health officials and medical organizations to implement targeted disease prevention and control. To our knowledge, this is the first study involving a retrospective analysis of ascariasis, enterobiasis and CE on a nationwide scale in Romania. It is also the first to use AutoTS technology for parasitic NTD.

## Author summary

Eastern and South-Eastern Europe is known to be severely affected by parasitic neglected tropical diseases (NTD) due to its tumultuous historical events of the past decades and to its uncontrolled socio-economic fluctuations. Romania is an example of such a South-Eastern European country that was known to have a high parasitic NTD burden after the fall of Communism in 1989 but has since made significant developmental improvements. However, there is scarce data regarding the incidences of parasitic NTD in Romania after its accession to the European Union in 2007. By using the ICD-10 dataset of Romania over the period 2008–2018, we performed a retrospective epidemiologic analysis of three of its most relevant parasitic diseases, ascariasis, enterobiasis and cystic echinococcosis (CE) and confirmed a downward trend strongly correlating with the country’s decreasing poverty rate. By employing a novel technology called automated time series machine learning we predicted the progress of these diseases for the ensuing two years of 2019 and 2020. Forecasted rates were observed to be constant. Such machine learning tools can help public health officials in adapting and improving targeted measures to combat parasitic NTD.

## Introduction

Neglected tropical diseases (NTD) are a group of communicable diseases caused by bacterial infections, viral infections or parasitic infestations [1,2]. NTD are “neglected” since they have been generally overlooked and typically have low prevalence rates in Western countries [3,4]. They relate to poverty, and they occur in areas that have inadequate healthcare, sanitation, and clean water supply. They also thrive in areas where people live in close proximity with animals and disease vectors [4]. Their incidence in Europe has been attributed mainly to travelers from endemic areas or asylum seekers [5].

When considering individual European regions, Eastern and South-Eastern Europe display high incidences of parasitic NTD [6,7]. Given the socioeconomic instability of these regions that resulted from the historical events of the past decades such as the fall of the Iron Curtain, the Revolutions of 1989 and the Balkan Wars, NTD were conditioned to thrive in such socially and economically destabilized settings [7,8]. Minimal government and veterinary supervision, with insufficient meat inspection, might have also contributed to the high incidence of neglected parasitic zoonoses [8–10].

The above conditions are widespread in Romania, a South-Eastern European country. Romania has many documented parasitic NTD, especially protozoa and helminths [8,11]. Only a few epidemiological studies have been conducted on parasitic diseases in Romania, most spanning from the early 1990s until about 2008. Nonetheless, intestinal parasitoses have emerged as an important public health issue due to their high incidence rates [7,11–13]. This is attributed to the cessation of large slaughterhouses and the persistence of the traditional “pig’s alms,” which involves backyard slaughtering and unsanitary conditions [14].

In addition to affecting people living in poor and unhealthy conditions, intestinal parasitic NTD affect the young population of Romania. The highest incidence was detected for ascariasis in children aged 0–14 years [12]. Ascariasis is caused by the roundworm *Ascaris lumbricoides* and is the most prevalent helminth infection worldwide [15]. Children tend to have relatively high worm burdens; they maintain the infestation rate by defecating indiscriminately in their environment and collecting infective eggs while playing [15,16]. Ascariasis causes approximately 60,000 deaths per year worldwide [17]. The disease can cause severe complications, such as intestinal obstruction, appendicitis, or peritonitis. Such conditions require hospitalization [18]. The prevalence of ascariasis in Romania varies markedly, between 4% and 69.1%; the last reported year was 2006 [12,13].

Another helminthiasis that has been widely spread both before and after the fall of Communism in Romania is enterobiasis. It is caused by the pinworm *Enterobius vermicularis* [13]. This NTD is one of the oldest and most prevalent intestinal parasites, affecting about 200 million people worldwide [19,20]. Enterobiasis is mostly asymptomatic. It can, however, lead to infections of the cervix, pelvis, urinary tract, and the peritoneum [21]. The prevalence of *E*. *vermicularis* infections of the gastrointestinal tract ranges from 4% to 28% [22]. Some authors even consider *E*. *vermicularis* to be one of the most important neglected causes of appendicitis [23]. Thus, severe forms of enterobiasis also require hospitalization with intensified diagnostics and therapy. Furthermore, the mean hospital stay for intestinal nematode infections in Romania has been calculated to range from 3 to 25 days [24]. The Romanian incidence of enterobiasis in children aged 0–14 years was reported to be even higher than ascariasis between between 1993 and 2006 [12]. Together with ascariasis, enterobiasis is one of the most commonly detected NTD in Romania; it accounts for up to 5.8% of positive test results for various parasites [13].

Lastly, *Echinococcus granulosus* is another hyperendemic helminth present in Romania [25]. This type of tapeworm is responsible for the NTD called “cystic echinococcosis,” which leads to the development of one or multiple cysts in the liver, lungs, or other organs [26]. In Romania, almost 50% of patients from birth to 19 years have hepatic or pulmonary affections [13]. Local epidemiological data indicate that at least one person in almost half of all Romanian localities has received surgery for cystic echinococcosis [27]. Moreover, Romania is listed to have one of the highest CE rates worldwide [28].

Since Romania’s accession to the European Union (EU) in 2007, stronger policies regarding health, safety and food standards have been implemented [29]. Although the health status of Romanians has improved and life expectancy at birth has increased by four years since 2000, major challenges still need to be addressed. These include substantial regional income-related disparities, control of infectious diseases and access to medical care [30,31]. Furthermore, after 2007 the distribution of only a few selected parasitic diseases has been analyzed, such as taeniosis and cysticercosis [8], cryptosporidiosis and giardiasis [32,33]. With CE having been analyzed only in a cross-sectional study design up to 2014 [34], there is an evident lack of new epidemiologic data after 2007 on a national level in Romania for the parasitic NTD, ascariasis, enterobiasis and CE. Since NTD are commonly found among the poorest populations and in the least developed countries of the planet and Romania is still exhibiting some of the highest yet constantly decreasing poverty rates in the EU [35], we hypothesized that the incidences of the above mentioned NTD have fallen after 2007. Despite having mostly asymptomatic courses, all three mentioned parasitic diseases do often lead to exacerbations that require hospitalizations. Being highly prevalent parasitic diseases, we stipulated that the hospitalization case rates of ascariasis, enterobiasis and CE would render a solid assessment of their epidemiologic distribution in the recent years in Romania.

In this study, we performed both a retrospective and a predictive time series analysis of these three majors helminthic NTD in Romania. For this purpose, we utilized the International Classification of the Diseases (ICD-10) dataset of Romania for 2008–2018 to assess the recent incidences of hospitalization of the three diseases at the regional NUTS 2 level [36]. By employing a novel machine learning (ML) technology called automated machine learning (AutoML) [37], we aimed to forecast the incidences of these parasitoses for the following two years, 2019 and 2020.

Machine learning can be used in healthcare as a diagnostic tool [38–40] or to promote clinical research [41–44] and to improve the efficiency of medical systems [45–47]. However, the demand for ML exceeds the expertise of healthcare providers who can effectively apply this technology [48]. AutoML circumvents this challenge by performing massive parallel processing and allowing users to build predictive models rapidly. By using automated time series ML, we recently predicted the incidences of the ten deadliest diseases in Romania as defined by the WHO [49]. Time series forecasting is mainly performed for infectious diseases, such as influenza [50–55]; hand, foot and mouth disease [56–59] and tuberculosis [60–62]. There is little forecasting of parasitic NTD in the current literature [63]. In addition, most time series analyses have employed only a few predictive models [49]. By contrast, AutoML on time series (AutoTS) tests and evaluates hundreds of models and allows selection of the most accurate model for a given time series dataset [64].

To our knowledge, this is the first study involving AutoTS for parasitic NTD and prediction of the monthly incidences of hospitalization of these diseases on a regional NUTS 2 level. The aims of this project were to evaluate the hospitalization incidences of the three selected NTD from 2008 until 2018 on a national scale in Romania and to forecast hospitalization cases using a highly accurate novel technology, in order to assist healthcare providers to improve their surveillance, to implement appropriate control plans and to allocate resources effectively in endemic regions.

## Materials and methods

### Ethics statement

This study was reviewed and approved by two ethics committees. The first was the committee of the National School of Public Health, Management and Professional Development (NSPHMPDB) in Bucharest, Romania (4854–04.11.2019 and DG286-22.01.2020). The second was the Medical Ethics Committee II of the Medical Faculty Mannheim, Heidelberg University (2019-873R) in Germany.

### Data selection and preparation

Starting in 2003, all hospitalized patients in Romania have been classified in a diagnosis-related group (DRG) database [65]. All Romanian hospitals report their DRG data monthly to the National School of Public Health, Management and Professional Development (NSPHMPDB) in Bucharest. Using the National DRG Database, we extracted time series datasets for a period of 11 years, from 2008 to 2018. These secondary datasets were extracted on a regional NUTS 2 level, according to the corresponding ICD-10 code for selected NTD provided by NSPHMPDB (S1 Table). The ICD-10 disease codes for ascariasis, enterobiasis and cystic echinococcosis were searched and validated using the WHO ICD-10 online application [66]. Only hospitalized cases for which targeted diseases were recorded as the main and secondary diagnosis were selected; the data was then aggregated into hospitalized cases per month per NUTS 2 region. More specifically, a single case was defined as an inpatient care of an individual with any severity, whose condition required admission to a hospital either as a direct consequence of the given parasitic infestation or who was found to have an asymptotic parasitic infestation. Data was prepared using Paxata in the DataRobot platform [67]. The secondary time series database is deposited online (https://www.synapse.org/#!Synapse:syn25870975/files/).

### Additional datasets

Incidences rates were calculated as monthly disease cases per 100,000 inhabitants [68,69] by dividing the total hospitalized cases per month by the total population for that month, for each Romanian NUTS 2 region. Results were then multiplied by 100,000. Monthly population data was obtained from Eurostat [70]. Another additional dataset employed for correlation analysis was “people at risk of poverty or social exclusion”, which was also extracted from Eurostat (S1 Fig). This variable represented the percentage of the population in Romania that fulfilled more than one of the following three criteria: (i) at risk of poverty, (ii) severely materially deprived, (iii) living in a household with very low work intensity [71].

### Descriptive and inferential statistics

Stata (Version Stata/IC 16, StataCorp, Texas, USA) was used for descriptive and inferential statistical analysis. For both its longitudinal and cross-sectional characteristics, the data used can be defined as panel data. Panel data comprises “n” entities with “T” observations through a time period “t”, and is commonly classified in short panels with large “n” and small “T”; or long panels with small “n” and large “T.” Another common classification distinguishes between datasets having balanced or unbalanced data. Datasets are balanced when all entities have data available for all time periods.

In our study, we worked with a long and balanced data panel dataset for each disease, with “N = n*T = 1056” observations resulting from “T = 132” (11*12 months) periods and “n = 8” total NUTS 2 regions. Both NUTS 2 regions and time period entities were consistent and not subject to change, with periods including data on monthly disease case rates and annual percentages of people at risk of poverty or social exclusion. We examined the differences between NUTS 2 regions and assessed the influence of the chosen poverty-related indicator on the monthly case rate. A one-way least squares dummy variable (LSDV) regression model was employed for this purpose. LSDV is a fixed-effect model used for panel regression. It provided an overview of the incidence rate of parasitic NTD by enabling us to create equations that included dummies for entities (NUTS 2 regions, in this instance) [72]. LSDV was chosen because such fixed-effect models can be employed to address potential omitted variables [73]. Furthermore, this fixed-effect model was preferred over a random-effect model as we assumed that the distribution of parasitic NTD is heavily influenced by time-invariant regional differences and there is a consensus among researchers to opt for the former model in such cases [74]. In the LSDV regression model, the dependent variable of interest was the monthly case rate of the selected NTD. The independent variable was the above-mentioned poverty indicator, henceforth termed “poverty rate.”

### Time series forecasting with automated machine learning

The methodology of time series forecasting with AutoTS has been previously described [49]. In short, each time series dataset is uploaded onto the AutoTS platform [75] and the appropriate forecasting target (e.g. “hospitalized cases”) is selected. A time frame is then set to define a derivation window (DW) to obtain descriptive features relative to the forecast point (FP). The FP is the time at which the prediction is made. 4, 6, 8, 10 and 12 months before the FP for each disease were used to empirically test DW. The derivation window that produced models having the smallest mean absolute percentage error (MAPE) was chosen (Table 1).

**Table 1:** Selected model performance validation based on holdout. After choosing the length of training data for the backtests, derivation window (DW) and the length of forecasted window (FW), models were compared and validated for each disease by the AutoTS platform. 2018 was chosen as holdout and the predicted values were compared to the actual values. Model selection was based on the mean absolute percentage error (MAPE). Other calculated estimators such as Gamma Deviance, root mean square error (RMSE), R-squared and the mean absolute error (MAE) are listed as well.

Disease	Months	Compared Models	Selected Model	MAPE	Gamma Deviance	RMSE	R-Squared	MAE
Ascariasis	DW = 12 FW = 24	24	Performance Clustered eXtreme Gradient Boosted Trees Regressor	52.1849	0.2487	8.6431	0.8188	6.2645
Enterobiasis	DW = 12 FW = 24	24	eXtreme Gradient Boosted Trees Regressor with Early Stopping (Gamma Loss)	41.5561	0.1707	8.3333	0.5620	6.4464
Cystic Echinococcosis	DW = 12 FW = 24	24	eXtreme Gradient Boosted Trees Regressor with Early Stopping (Gamma Loss)	40.0085	0.1990	10.0390	0.8231	5.9901

Next, a forecast window (FW) of 24 months was used for each disease. The FW defines the range of future values chosen to be predicted relative to FP. FW greater than 24 months have been avoided to reduce decaying accuracy in forecast across time. After defining the above-mentioned modeling settings and target, a model fitting procedure of preprocessing, algorithms and post-processing steps was performed by the AutoTS tool (S2 Fig). The AutoTS platform simplifies model development by performing a parallel heuristic search for the best model or ensemble of models; this search is based on the characteristics of the data and the prediction target. During the modeling process, many independent challenger models are developed. Each model’s performance is assessed by employing out-of-time validation (OTV), which allows the selection of specific time periods to test the model stability, creating data backtests [76]. Backtests are employed to reduce overfitting of models. In this instance, three backtests with a validation length of 1 year were used for each time series dataset (S3 Fig). In addition to OTV partitioning, a holdout sample to further test out-of-sample model performance was used. The year 2018 was chosen as the holdout partition. The details of how these models were built and how they perform are ultimately exposed, enabling the selection of the best model (Table 2).

**Table 2:** Exemplary listing of model performances calculated by the AML tool for ascariasis. The AutoTS tool automatically creates and selects time series features in the modeling data and will automatically detect whether or not a project’s target value is stationary (that is, whether the statistical properties of the target are constant over time). If the target is not stationary, the AutoTS tool attempts to make it stationary by applying a differencing strategy prior to modeling. This improves the accuracy and robustness of the underlying models. This differencing strategy includes calculating difference of the time series itself with either the most recent value (latest) or the average baseline as seen in the column ’Feature List and Sample Size’. The optimization metric used was MAPE (mean absolute percentage error). The ’All Backtests Score’ represents the average of all backtests. The model types considered during the model selection process included the following 8 out of 24 models, which are sorted by the holdout score. The Performance Clustered eXtreme Gradient Boosted Trees Regressor model was further used for prediction since it rendered the best MAPE score.

Model Name	Backtest 1 Score (MAPE)	All Backtests Score (MAPE)	Holdout Score (MAPE)	Feature List and Sample Size
**Ascariasis**				
Performance Clustered eXtreme Gradient Boosted Trees Regressor	43.1241	41.0072	52.1849	With Differencing (average baseline) 3 years • 11 months • 1 second
eXtreme Gradient Boosted Trees Regressor with Early Stopping (Gamma Loss)	44.5819	42.1621	54.6906	With Differencing (average baseline) 3 years • 11 months • 1 second
eXtreme Gradient Boosting on ElasticNet Predictions (Gamma Loss)	44.0895	41.3165	55.8184	Reduced Features 3 years • 11 months • 1 second
Performance Clustered eXtreme Gradient Boosted Trees Regressor	42.3463	41.7421	56.2874	With Differencing (latest) 3 years • 11 months • 1 second
Light Gradient Boosting on ElasticNet Predictions (Gamma Loss)	43.1089	41.9593	57.1755	With Differencing (latest) 3 years • 11 months • 1 second
Performance Clustered eXtreme Gradient Boosting on Elastic Net Predictions	43.8631	42.2386	58.3096	No Differencing 3 years • 11 months • 1 second
Performance Clustered eXtreme Gradient Boosting on Elastic Net Predictions	43.6917	41.4166	58.6925	With Differencing (latest) 3 years • 11 months • 1 second
AVG Blender	44.9861	42.0284	58.7932	Multiple Feature Lists 3 years • 11 months • 1 second

Finally, after selecting the desired model, we obtained predictions by allowing the model to estimate values of hospitalized cases per NUTS 2 hospital region. This analysis was performed for each of the 24 months in the forecast window, namely 12 months in 2019 and 12 months in 2020.

## Results

### Retrospective analysis of hospitalization rates for ascariasis, enterobiasis and cystic echinococcosis in Romania over the period 2008–2018

The 10th revision of the International Classification of Diseases (ICD-10) is an internationally implemented medical classification system designed by the WHO [77]. It is employed for classifying diseases, symptoms, types of injuries and even medical procedures. It is used by healthcare providers for billing and reimbursement purposes and by researchers as an important tool for disease surveillance [78]. To assess the incidence rates of hospitalization and evaluate regional differences of ascariasis, enterobiasis and cystic echinococcosis, we extracted the ICD-10 codes of these three parasitic NTD from the national ICD-10 dataset of Romania for 2008–2018 and grouped all cases into eight NUTS 2 regions of Romania.

#### Ascariasis

**Fig 1:**
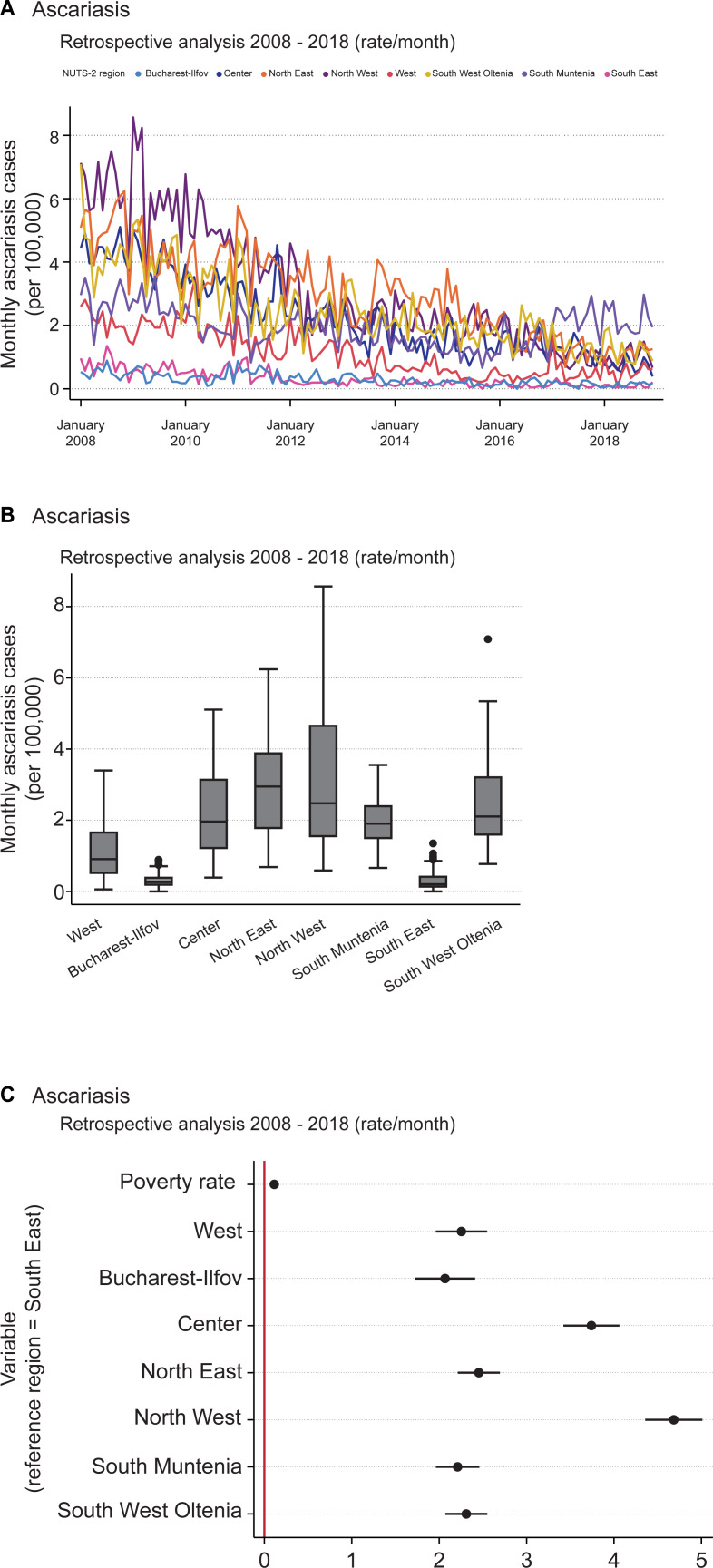
Retrospective analysis of ascariasis incidence rates of hospitalization over the period 2008–2018. (A) Monthly incidence rates (cases per 100,000 inhabitants) of patients hospitalized due to ascariasis over the period 2008–2018. (B) Box plot of monthly ascariasis cases (per 100,000) by NUTS 2 region over the period 2008–2018. The horizontal black lines depict the median values; the boxes go from the 25th to the 75th percentile of each region’s distribution of values; further vertical extensions refer to adjacent values; dots are outliers. (C) Regression coefficient plot of LSDV model created for monthly ascariasis incidence rate. The horizontal bold lines represent the width of 95% confidence interval for the parameters. Variables are not significant in case of an intersection of the confidence interval with the vertical reference red line at 0.

The retrospective analysis of monthly ascariasis hospitalization incidence rates indicated an evident decline in cases for most regions from 2008 to 2018 (Fig 1A). The northern NUTS 2 regions, namely North West and North East, had the highest case rates until 2015. By contrast, South Muntenia showed constant rates throughout the observed period and ultimately displayed the highest rates among all regions in 2017 and 2018. Interestingly, Bucharest-Ilfov and South East recorded notably few hospitalization cases, with the minimum (zero) monthly cases in South East during September 2015. The maximum – approximately 8.6 monthly cases per 100,000 – was observed in North West in January 2009. The general mean ascariasis incidence rate was approximately 1.8 (Fig 1A, Table 3). The deviations between the actual monthly incidence rate and the region’s average monthly incidences are depicted as “within” values in Table 3. Regarding ascariasis, the maximum value of 7.275 was notably higher than the minimum value of -0.705. This result suggests a rapid decline in the monthly hospitalization incidence rate, moving towards more consistent and lower incidence rates. However, the deviation between the monthly incidence rates across regions, as shown by “between” values (1.114), was nearly equal to the “within” deviation (1.112) for the observed period (2008–2018). The results are shown in Table 3. That is, when incidence rates are randomly selected from two regions, the difference between those two rates is similar to the differences in incidence rates for the same region across two randomly selected months.

**Table 3:** Summary of means, deviations, minimums, maximums, and total observations of ascariasis, enterobiasis and cystic echinococcosis. “Mean” represents the overall mean value of incidence rates. “Overall” deviation represents the deviation over time and NUTS 2 regions. “Between” variation represents the deviation across NUTS 2 regions (time-invariant). “Within” variation represents the deviation of incidence rates over time (time-variant). Total number of observations “N = n*T = 1056” results from total time periods “T = 132” (11*12 months) and total NUTS 2 regions “n = 8”.

		Mean	Standard Deviation	Min	Max	Observations
**Ascariasis**	**overall**	**1.808**	**1.525**	**0**	**8.567**	**N = 1056**
**Monthly incidence rates**	**between**		**1.114**	**0.292**	**3.100**	**n = 8**
	**within**		**1.112**	**-0.705**	**7.275**	**T = 132**
**Enterobiasis**	**overall**	**1.582**	**1.182**	**0**	**7.534**	**N = 1056**
**Monthly incidence rates**	**between**		**0.856**	**0.650**	**3.182**	**n = 8**
	**within**		**0.868**	**-0.719**	**6.747**	**T = 132**
**Cystic Echinococcosis**	**overall**	**1.146**	**1.391**	**0**	**8.027**	**N = 1056**
**Monthly incidence rates**	**between**		**1.359**	**0.332**	**4.433**	**n = 8**
	**within**		**0.563**	**-2.034**	**4.740**	**T = 132**

The boxplot of monthly ascariasis incidences of hospitalization throughout the NUTS 2 regions clearly indicates the regional differences in incidence rates (Fig 1B). In Bucharest-Ilfov and South East, the medians are notably low and the interquartile ranges are relatively small. The remaining regions show much wider spreads. North East and North West display the highest medians and widest spreads of all ascariasis cases; these results indicate the most substantial changes in incidence rates during 2008–2018.

To statistically examine the observed regional differences and to evaluate whether the poverty rate correlated with the calculated incidence rates for ascariasis, we used an LSDV model. The dummy variable region “South East” was excluded and served as the reference region, with the constant -5.111 being the baseline estimate (Y-intercept) of this region (Fig 1C, S2 Table). Thus, the coefficient 2.254 calculated for the West region was used to estimate the deviation of the intercept of the West region from the baseline of -5.111. This was computed as “2.857 = -5.111 + 2.254”. To form regression equations for each NUTS 2 region, the coefficient of 0.113 obtained for the poverty rate was included (S2 Table). The deviations of the intercepts were all statistically discernable from zero (p < 0.01). The results indicate that regional differences did exist (S3 Table).

#### Enterobiasis

**Fig 2:**
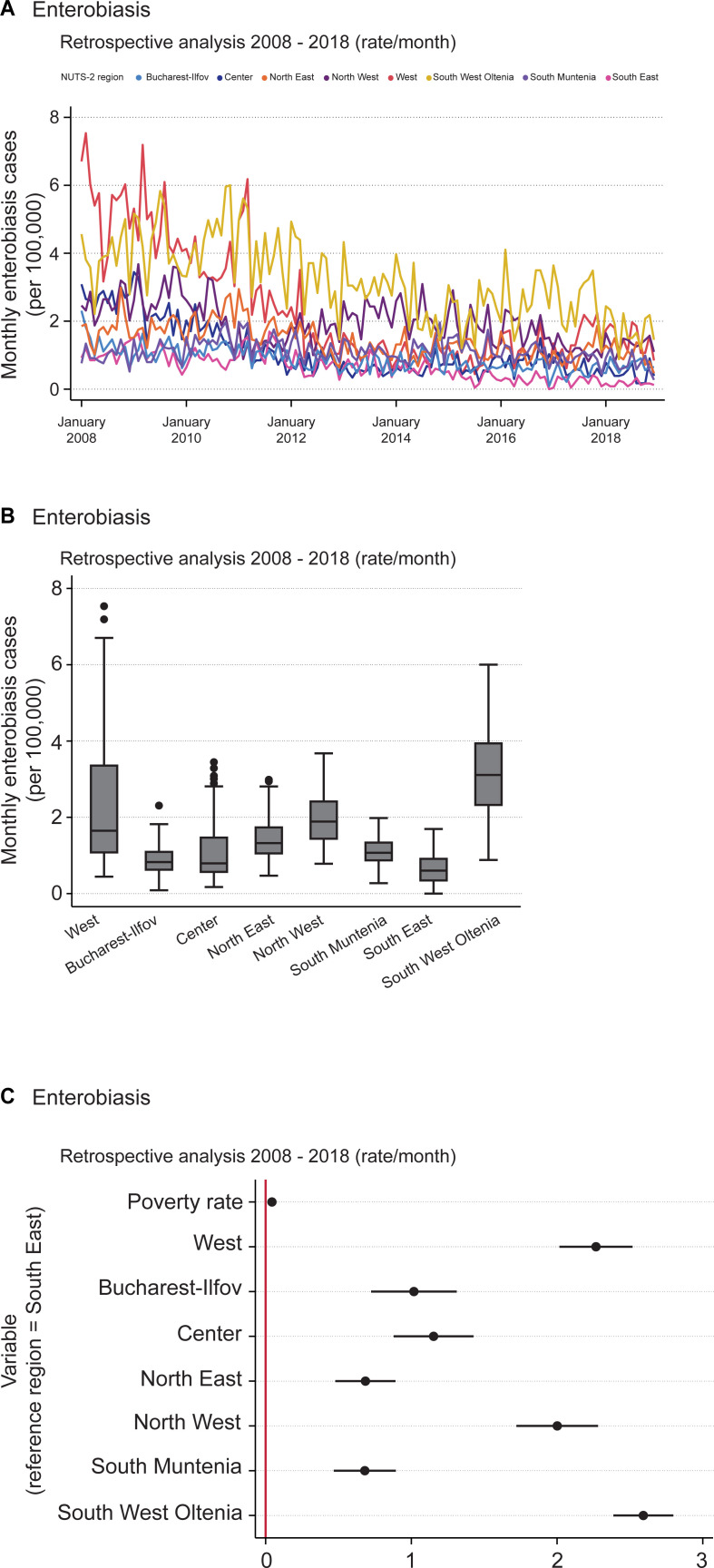
Retrospective analysis of enterobiasis incidence rates of hospitalization over the period 2008–2018. (A) Monthly incidence rates (cases per 100,000 inhabitants) of patients hospitalized due to enterobiasis over the period 2008–2018. (B) Box plot of monthly enterobiasis cases (per 100,000) by NUTS 2 region over the period 2008–2018. The horizontal black lines depict the median values; the boxes go from the 25th to the 75th percentile of each region’s distribution of values; further vertical extensions refer to adjacent values; dots are outliers. (C) Regression coefficient plot of LSDV model created for monthly enterobiasis incidence rate. The horizontal bold lines represent the width of 95% confidence interval for the parameters. Variables are not significant in case of an intersection of the confidence interval with the vertical reference red line at 0.

The highest enterobiasis monthly incidence rates of hospitalization were found in the western regions, namely West, South West Oltenia and North West. The first two regions displayed a clear decline, with wide spreads between 2008 and 2018 (Fig 2A and 2B). Compared with ascariasis, a slightly more oscillating pattern was observed. The remaining regions also showed a decline, but not nearly as steep as that of West and South West Oltenia. Bucharest-Ilfov and South East displayed the lowest incidence rates.

The overall mean enterobiasis incidence rate was approximately 1.5 cases each month for every 100,000 people. The minimum was zero monthly cases, reached in South East in December 2016, and the maximum was 7.5 monthly cases in the West in February 2008 (Fig 2A, Table 3). Similar to the scenario regarding ascariasis, there was a striking pattern regarding the difference between the highest and lowest deviations of monthly incidence rates. We dubbed this difference the “within” value. The highest monthly incidence rate deviation was 6.747, and the lowest was -0.719.

Furthermore, the “between” deviation in monthly incidence rates – namely, across regions – was similar to the “within” deviation over the observed period. The deviation between regions was 0.856 and the “within” deviation was 0.868 (Table 3). Using South East as a reference region, we created a coefficient plot (Fig 2C) and regression equations for monthly enterobiasis cases (S2 Table). Once again, the intercepts were all statistically discernable from zero (p < 0.01). This result suggests the existence of regional differences and a strong correlation between the disease and poverty rates (S3 Table).

#### Cystic echinococcosis

**Fig 3:**
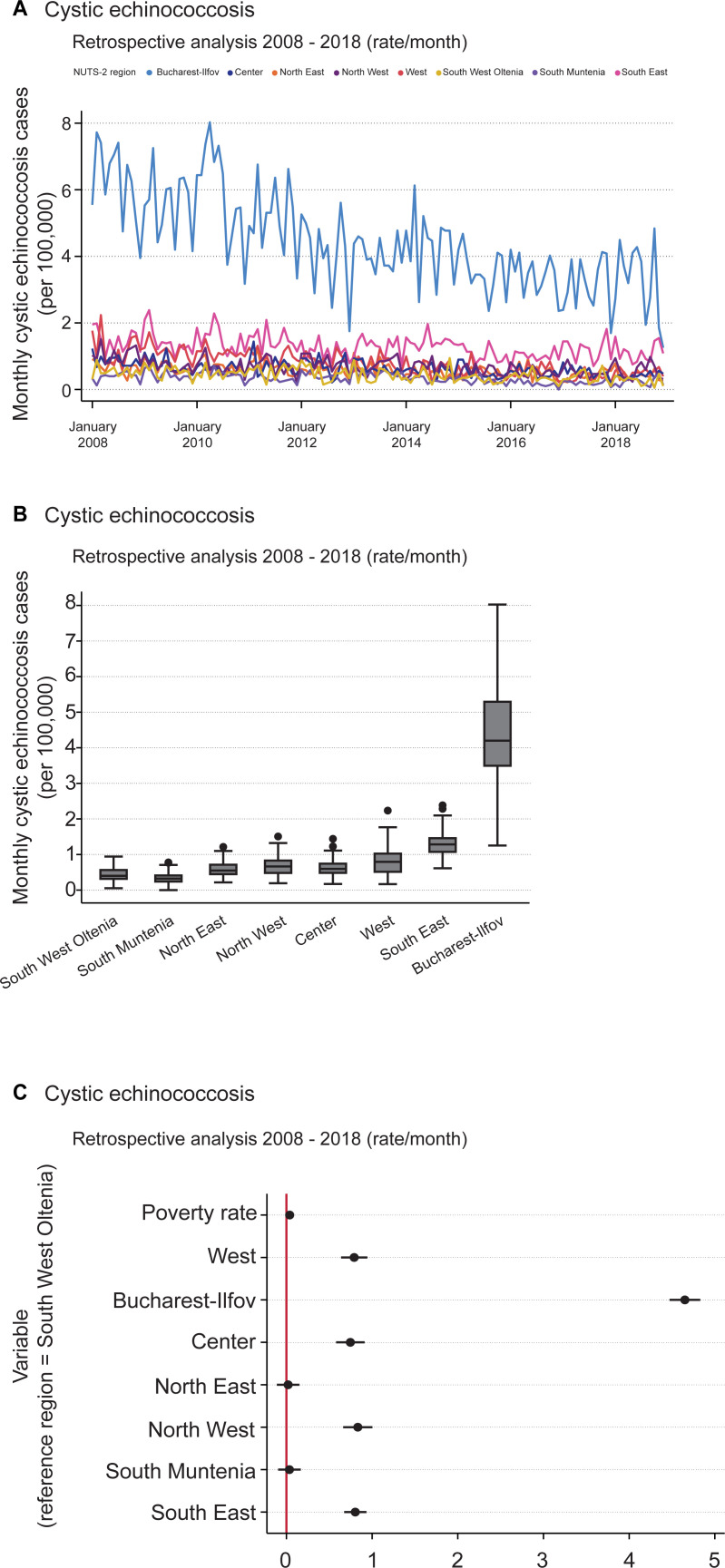
Retrospective analysis of cystic echinococcosis incidence rates of hospitalization over the period 2008–2018. (A) Monthly incidence rates (cases per 100,000 inhabitants) of patients hospitalized due to cystic echinococcosis over the period 2008–2018. (B) Box plot of monthly cystic echinococcosis cases (per 100,000) by NUTS 2 region over the period 2008–2018. The horizontal black lines depict the median values; the boxes go from the 25th to the 75th percentile of each region’s distribution of values; further vertical extensions refer to adjacent values; dots are outliers. (C) Regression coefficient plot of LSDV model created for monthly cystic echinococcosis incidence rate. The horizontal bold lines represent the width of 95% confidence interval for the parameters. Variables are not significant in case of an intersection of the confidence interval with the vertical reference red line at 0.

Contrary to ascariasis and enterobiasis, CE hospitalization incidence rates were highest in Bucharest-Ilfov; South East had the second highest rates, but with a substantial gap (Fig 3A). All the other regions shared similar low incidence rates, with a median far below that of Bucharest-Ilfov (Fig 3B). Bucharest-Ilfov showed a decline in incidence rates from 2008 to 2018. In the remaining regions, no clear negative or positive tendencies were observed.

The overall mean for monthly CE incidence rates was approximately 1.1 cases per 100,000 people. The minimum was zero monthly cases in South Muntenia in December 2016, and the maximum was almost 8 monthly cases per 100,000 people in Bucharest-Ilfov during April 2010 (Fig 3A, Table 3). Regarding the summary panel data (Table 3), the difference in “within” deviation values was smaller for CE than for ascariasis or enterobiasis.

The deviation in monthly incidence rates across the regions (“between” values) was more than twice as high as the “within” values for 2008–2018. The “between” value was 1.359 and the “within” value was 0.563. That is, when randomly picking incidence rates from two regions, the incidence rate difference is expected to be more than twice as high than the difference from two incidence rates of the same region in two randomly selected months.

The coefficient plot (Fig 3C) and regression equations (S2 Table) for CE were obtained with the same computing technique and using South West Oltenia as a reference region. For the regions North East and South Muntenia, deviations from the intercept were not statistically discernable from zero, whereas all remaining regions and the poverty rate did reveal a statistical significance (S3 Table).

### Time series forecasting for 2019 and 2020

**Fig 4:**
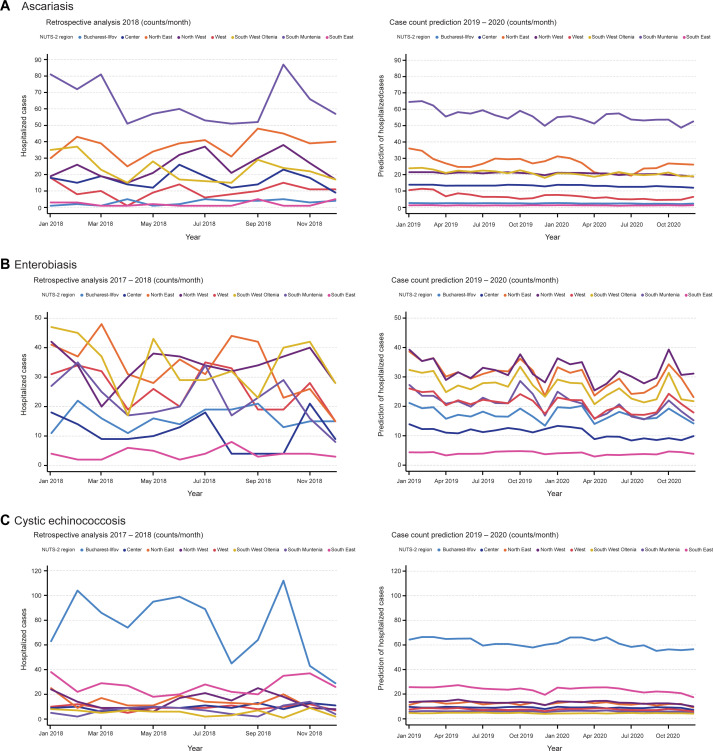
Prediction of hospitalization cases of ascariasis, enterobiasis and cystic echinococcosis for 2019 and 2020. Comparison between actual hospitalization cases in the year 2018 (left side) and predicted disease cases for the years 2019 and 2020 (right side). Hospitalized cases of (A) ascariasis, (B) enterobiasis and (C) cystic echinococcosis were plotted on a monthly basis per NUTS 2 region against the predicted values.

Since the total monthly population was unknown for the predicted future time points during the study period, AutoML forecasting was performed with time series datasets that comprised the total numbers of monthly hospitalized cases per NUTS 2 region. To facilitate visualization and comprehension, hospitalization case counts for each of the analyzed NTD were plotted on a monthly basis for 2018 (Fig 4, left panel). This year was also chosen as the holdout partition, which was excluded by the AutoTS platform from the time series datasets; the 2018 data was used only to verify the models (Table 1).

The best-performing models were selected based on the mean absolute percentage error (MAPE). For ascariasis, the selected model was the Performance Clustered eXtreme Gradient Boosted Trees Regressor, while the eXtreme Gradient Boosted Trees Regressor with Early Stopping (Gamma Loss) was chosen for both enterobiasis and CE. Extreme gradient boosting is an efficient version of gradient boosting ensemble machine learning algorithm, which has been optimized and tweaked for fast runtimes and predictive accuracy [79].

The course of the 2018 curves showed some degree of intersections (Fig 4, left panel). Predominant parallel curve progressions were noticeable for the predicted years, 2019 and 2020 (Fig 4, right panel). Most case counts seemed to be constant throughout the predicted 24 months, with no striking declines or rises. Total predicted hospitalized case counts were validated against the actual hospitalized cases extracted from the National DRG Database for the years 2019 and 2020 (S4 Fig). While ascariasis and CE had alternating, but similar case counts; enterobiasis did indeed reveal fewer actual cases, but a parallel progression to the predicted cases. Furthermore, the actual cases showed similar monthly fluctuations as the 2018 curves (Fig 4, left panel) and the predicted curves revealed steadier progressions. Next, the constancy of hospitalized cases was confirmed for 2019 and the first two months of 2020. Starting with March 2020 all hospitalizations of the analyzed parasitoses showed a massive drop and continuously low case counts for the rest of 2020 (S4 Fig).

Closer inspection indicated that indeed a slight drop in hospitalization counts had occurred for ascariasis (Fig 4A). Here, South Muntenia and North East remained the regions with the highest counts, whereas North East almost halved its hospitalization episodes for mid-2020 (April and July) compared to 2018. North West, which had the highest case rates between 2008 and 2011 (Fig 1A), had the least fluctuations, with about 20 cases at any point in the predicted years.

The prediction of enterobiasis indicated that North West and North East were the regions with the highest monthly case numbers (Fig 4B). A seasonal pattern was observed, with spikes in October followed by dips in December and April. The predicted case counts for CE showed a stable course, with Bucharest-Ilfov and South East remaining the regions with the highest and second highest numbers of cases, respectively (Fig 4C). Another small dip occurred in December throughout most in regions for cases of CE.

## Discussion

Parasitic diseases have accompanied humankind throughout its existence. While medical advances and public health policies in recent decades have reduced the transmission and severity of parasitic infestations, it remains almost impossible to achieve eradication [80]. Deciphering the fundamental processes of parasitic organisms through biomedical research is essential. In addition, disease surveillance using epidemiological data is an efficient approach to observing, predicting, and controlling transmission. The analysis of reported data can provide insight into diseases’ contributing factors; the findings can also indicate the efficiency of public health strategies.

In this study, we calculated the incidence rates of hospitalization for three parasitic NTD: ascariasis, enterobiasis and cystic echinococcosis. We performed predictions at the regional level of Romania using the national ICD-10 dataset for a period of 11 years. An overview of the progression curves indicated a decline in incidence rates of hospitalization for all three NTD during the analyzed period. This case reduction in all NUTS 2 regions was most noteworthy for ascariasis. South Muntenia emerged as a potential current center of hyperendemicity; the same pattern occurred in 2017–2018 as in the predicted period 2019–2020. The Ascaris species has been included in the European food-borne parasite prioritization list to help improve targeted surveillance [6]. However, there is scant epidemiological data available on the distribution of ascariasis in Romania, and it is outdated. Prevalence estimates range widely, from 4% to 82% [24]. One reported prevalence rate for ascariasis, of 35.7%, was calculated based on only 42 investigated patients at a single hospital; such results are not generalizable to the whole of Romania [24]. A different study on the epidemiology of ascariasis for 1993–2006 in Timis County, which is part of the NUTS 2 region West, reported higher but steadily declining case rates. The latter study supports our observation that ascariasis incidence is decreasing [12]. Interestingly, that study also found relatively high case rates in rural areas. Similarly, in our study, the NUTS 2 region of Bucharest-Ilfov – comprising mainly the capital of Romania, Bucharest – had one of the lowest counts. The rise in case rates in South Muntenia, starting in 2017, remains to be elucidated. The annual incidence in the southern neighboring country of Bulgaria was estimated to be 7.84 per 100,000 [81]. This is higher than the mean incidence rate for ascariasis in Romania in the analyzed period. Bulgaria is a top travel destination for Romanian tourists, with more than 1 million Romanians having visited Bulgaria in 2017 and 2.1 million in 2019 alone [82,83]. Next to free transit and socio-economic exchanges at the border between southern Romania and northern Bulgaria, there are also different regional and European health programmes that promote cross-border cooperation [84–87]. Thus, we speculate that Bulgaria′s high case counts and its proximity to South Muntenia might serve as a potential explanation for the high incidence rates of hospitalization observed in this southern Romanian region.

The rate of people at risk of poverty or social exclusion has declined in all NUTS 2 regions during the 2008–2018 period. Therefore, a positive correlation between the poverty rate and the analyzed NTD in Romania seemed like a reasonable hypothesis that this study’s regression model and computed equations added further arguments to. The insertion into the equations of the current poverty rate allowed for a rough indication of the monthly NTD case rate at the regional level. For ascariasis, the goodness-of-fit measure, R-squared (0.579), indicated that additional time-invariant variables might need to be investigated to understand the disease’s epidemiology in Romania.

Like ascariasis, enterobiasis showed a general tendency towards lower case rates. The regions of West and South West Oltenia experienced the most notable reduction in the incidence rate of hospitalization for 2018, compared with 2008. Furthermore, a significant correlation to poverty rate was evident. Initially considered not to be associated with socioeconomic or cultural factors [88], enterobiasis has more recently been classified as a poverty-related parasitic disease [7,89]. Data from the Romanian County of Timis for 1993–2006 indicated a mean annual incidence of 777 cases per 100,000 inhabitants, without significant variation over time or for urban versus rural populations [12]. The discrepancy between our calculated mean incidence rate and the one from the County of Timis could have arisen from different data sources. While we examined only hospitalized cases, the local study in Timis used data from general practitioners [12]. Moreover, most enterobiasis cases are known to have an asymptomatic course, thus generating large research gaps – as reflected in the widely differing prevalence estimations [90,91].

Research in other countries suggested high prevalence rates in rural and suburban environments [92]. We could partially confirm this pattern, with low incidence rates noted for Bucharest-Ilfov. Contrary to the retrospective incidence rate reduction, we predicted a stable course for future enterobasis cases of hospitalization. Our analysis predicted a peak in enterobiasis hospitalization rates during October 2019 and October 2020. This prediction was confirmed by recent findings, which suggests a seasonal pattern, with high disease frequencies in winter months [93,94].

*Echinococcus granulosus* is the second-most prioritized foodborne parasite in Eastern Europe [6]. Romania was shown to be one of the most affected countries in the region [34,95]. While CE often exhibits no symptoms, due to the slow development of echinococcus cysts, severe forms with affections of the liver and the lung also require hospital treatment. This has led for several studies to determine the incidence of CE based solely on hospitalization episodes. CE incidences calculated with hospital records have already been published for countries such as Iran, Egypt, or Spain [96–98]. Similarly, hospitalization case rates were evaluated for Western Romania between 2007 and 2017 [28]. However, the true incidence of cystic echinococcosis in Romania might be even higher due to underreporting in European statistics. In 2013, for example, 55 CE cases were reported for the whole country; while one hospital in Bucharest recorded 104 cases [95]. Interestingly, we observed a higher rate of CE in Bucharest-Ilfov than all other NUTS 2 regions. Similar to the development of ascariasis and enterobiasis cases, as the poverty rate declined from 2008 to 2018, the monthly CE incidence rates of hospitalization also showed a decline. We could predict this negative trend with the regression model. The relatively high goodness-of-fit measure for our model (0.852) might indicate a high correlation of poverty rate and CE. However, other time-invariant factors could potentially account for the trend and for the high incidence rates in the capital of Romania.

A recent systematic review provided further insight regarding the epidemiological distribution of *Echinococcus granulosus* infection in human and domestic animal hosts in Romania [95]. The mean human annual incidence of CE was estimated at 5.70 cases per 100,000 inhabitants for 2000–2010 in the major socio-economic NUTS 1 region RO1. This region comprises North West and Center. Another estimate was 4.39 cases per 100,000 inhabitants for 1991–2008 in the NUTS1 region RO4, comprising South West Oltenia and West [95]. In contrast, our calculated CE incidence rate of hospitalization was higher in the macro-region RO1 in 2010.

The above-mentioned review also considered dog populations in Romania, since direct contact with contaminated dogs is known to facilitate transmission of *Echinococcus granulosus* to humans [99]. Romania was known to have many stray dogs, particularly in urban environments like Bucharest [100]. This led to an initiative by the Bucharest city hall to reduce stray dog numbers. Between October 2013 and January 2015, of the estimated initial 64,704 stray dogs in Bucharest, more than half of that number were euthanized and many other dogs were adopted or taken to shelters [101]. Moreover, Romania implemented a program that consisted of registration of all owned dogs, stray dog control and spaying of bitches [102]. For the period 1956–1992 the average prevalence of *E*. *granulosus* infections in dogs was of 21.6% (range 0–83%) [27]. Afterwards, higher prevalence was reported in 1997 in southern Romania with 75% of shepherd dogs and 6–87.5% of stray dogs infected [103]. Another report stated that the prevalence *E*. *granulosus* infections in dogs from urban areas was about 4.3% between 2011 and 2012 [104]. The vast drop in stray dog populations from 2013 to 2015 and the nation-wide control and periodical treatment of dogs could thus help to explain the decline in CE cases. Our predictions, however, showed stable CE disease rates for 2019 and 2020, requiring further health policies to combat other transmission routes of *Echinococcus granulosus*.

Our study renders a systematic depiction of the national incidence rates of hospitalization of ascariasis, enterobiasis and cystic echinococcosis in Romania after the country’s accession to the European Union. It is important to emphasize that hospitalization data may strongly differ from national disease surveillance data. While such hospitalization datasets could serve as better disease monitoring sources for specific medical conditions [105], national disease registries are still generally preferred by epidemiologists as more distinct and unbiased datasets [106]. Therefore, there might be differences in the magnitude of incidence rates and associated trends, when comparing our results to other studies based on surveillance data. However, it is the first study, to our knowledge, to perform AutoTS predictions for parasitic NTD. The accuracy of the selected prediction models was not as high as in previous studies [49]. Comparison to the actual cases revealed either a parallel monthly progression for enterobiasis, or equally high, but alternating case counts for ascariasis and CE. The differences in model accuracies can be attributed to limited training data (i.e. disease counts). The training data could be increased by adding ambulatory treated and asymptomatic patient groups. Moreover, asymptomatic hospitalized patients, who were not admitted to the hospital due to parasitosis or were not tested for parasitic infestations, but carried a parasitic disease, also represent another missing group from the analyzed dataset. However, there is currently no systematic data acquisition for both ambulatory and inpatient treated patients; asymptomatic patients are not even recorded in medical datasets because most do not seek medical treatment. We believe that a novel re-evaluated medical data acquisition method to include all these patient groups would contribute to machine learning analysis and more generally to epidemiological studies. Nevertheless, our predicted results can be considered by public health officials when adopting public health policies to better control these NTD.

Another limitation was the characteristic of NTD being commonly found within marginalized communities. The targeted populations are also the ones with potentially inequitable access to health care services, as is the case with Roma populations in Romania [107]. Bias caused by people moving to other areas for hospitalization was not taken into consideration, since the extracted secondary dataset contained hospital related and not patient related case counts. Moreover, hospitalization cases of the National DRG Database are counted only upon discharge, meaning that a few long-term hospitalized cases might have missed from the database during the study period. Another limiting factor could be insufficient knowledge among physicians about NTD. This point might have led to erroneous reporting or wrong classification of the diseases. For example, in a study on trichinellosis in Italy involving hospital discharge records from 2005 to 2016, 70.6% of the records were wrongly reported. Some of the reasons included the absence of clarity between the two parasitic diseases of trichiasis and trichinellosis [108]. Insufficient training of professional healthcare coders could thus lead to underreporting in such datasets. Moreover, the fixed-effect regression used in our analysis accounted for time-invariant variables. It did not account for time-variant variables, such as climate data, educational parameters or livestock numbers–all of which might have been potential confounders or effect modifiers. Another unaccounted variable represented pandemics. The first confirmed case of Severe Acute Respiratory Syndrome Coronavirus 2 (SARS-COV-2) was confirmed in Romania on February 26, 2020 [109]. By March 25, 2020 Romania instituted a military curfew with enhanced restrictive measures which led, among others, to a drastic reduction in pharmacy or hospital visits [110]. Furthermore, some hospitals discharged their hospitalized patients and were only admitting SARS-COV-2 infected patients in the context of countermeasures against the coronavirus disease 2019 (COVID-19) pandemic [111,112]. Sadly, due to late responses and insufficient preparations, some hospitals experienced quick SARS-COV-2 infection rates among their staffs leading to quarantine of these hospitals [110]. Thus, in 2020 hospital admissions showed an almost 40% reduction when compared to the previous years [113]. All the above mentioned COVID-19 related events were highly probable to have contributed to the observed drop in actual hospitalization cases for ascariasis, enterobiasis and CE starting with March 2020.

In conclusion, the retrospective and forecasted results presented here can be used to implement public health measures or to upgrade diagnostic and therapeutic procedures in specific regions. More specifically, reinforced eradication and control strategies are necessary for ascariasis in South Muntenia and CE in Bucharest-Ilfov since cases there will not continue to fall despite the decreasing trend from 2008 to 2018. Furthermore, all NUTS 2 regions with the exceptions of South East and Center should expect higher hospitalization cases of enterobiasis during the month October. In such cases, preventive measures should be undertaken consisting of improvement of sanitation, preparation of antiparasitic drug supply or health education. Such targeted actions could help to further decrease the incidence of the analyzed NTD.

## Supporting information

S1 Table Listing of ICD-10 codes selected for data extraction and preparation from the whole ICD-10 dataset of hospitalized patients in Romania during the period 2008–2018. Specific ICD-10 codes encoding either ascariasis, enterobiasis or cystic echinococcosis were selected according to the “ICD-10_AM diagnoses and procedures list” provided by the National School of Public Health, Management and Professional Development (NSPHMPDB) from Bucharest, Romania.
(DOCX)

S2 Table **Regression equations for (A) ascariasis, (B) enterobiasis and (C) cystic echinococcosis.** Reporting of regression equations for LSDV with a set of group dummy variables. The sets of group dummy variables were created to be able to compute regression equations that are specific to the NUTS 2 region. Each of the regions intercepts stands for the deviation of its group specific intercept from the intercept of the reference region. Example: To compute an approximation of the monthly ascariasis incidence rate in South East, the poverty rate can be inserted into the equation to obtain an approximation according to the fixed effects model used. In case of a poverty rate of 50%, a monthly ascariasis case rate of 0.539 (per 100,000) would be obtained.
(DOCX)

S3 Table **Regression tables for (A) ascariasis, (B) enterobiasis and (C) cystic echinococcosis.** The regression tables were computed with Stata’s “xi” command, which converts categorical variables into dummy or indicator variables when fitting a model. The dependent variable, i.e. the monthly incidence rate of hospitalization of the respective disease, is measured as cases per 100,000. The reference region was chosen so that for the respective disease the coefficients of the categorical variables, the NUTS 2 regions, would be positive. A higher coefficient of a region would indicate a higher baseline case rate and vice versa. Therefore, the reference region accounts for the lowest baseline incidence rate. Next, a positive coefficient on the independent variable “poverty rate” indicates a positive correlation with the dependent variable and vice versa. Both R-squared values and the F-Test provide goodness-of-fit measures.
(DOCX)

S1 Fig People at risk of poverty or social exclusion by Romanian NUTS 2 regions, as obtained from Eurostat [71]. X-axis represents the percentage of total population of people at risk of poverty or social exclusion. Y-axis represents the survey year.
(EPS)

S2 Fig **Model development workflow process (model blueprint) for (A) ascariasis, (B) enterobiasis and cystic echinococcosis.** A blueprint represents the end-to-end procedure for fitting the model, including any preprocessing steps, algorithms, and post-processing. It illustrates the many steps involved in transforming input predictors and targets into a model. Each node in a blueprint can represent multiple steps. The following elements connect to visualize the blueprint: “Ordinal encoding of categorical variables”, “Missing Values Imputed”, “Extract Forecast Distance Feature”, “Naive Predictions as Offset”, “Performance Clustered eXtreme Gradient Boosted Trees Regressor” (A) or “eXtreme Gradient Boosted Trees Regressor with Early Stopping (Gamma Loss)” (B), and “Text fit on Residuals (L2 / Gamma Deviance)”. The performance metric used for the projects were Gamma Deviance (shown in the blueprint), as well as MAPE, RMSE, R-squared and MAE. The projects included a total of 1053–1055 observations.
(EPS)

S3 Fig Representation of model development procedure for all employed NTD. The AML platform used 3 backtests with a validation length of 12 months, as well as a holdout fold with start date 2017-12-01 and end date 2018-12-01 for additional testing. This dataset is used to verify that the final model performs well on data that has not been touched throughout the training process. Grey denotes the available training data, blue denotes the validation partition, and green denotes the holdout sample.
(EPS)

S4 Fig Prediction validation. Comparison between predicted total hospitalized cases of ascariasis (A), enterobiasis (B) and cystic echinococcosis (C) and actual total hospitalized cases extracted from the National DRG Database for the years 2019 and 2020. All hospitalized cases in Romania (actual values) were plotted on a monthly basis against the predicted values calculated by the chosen model (predicted values).
(EPS)

## Abbreviations

ICD-10: International Classification of Diseases, Tenth Revision
NUTS: Nomenclature of Territorial Units for Statistics
WHO: World Health Organization
