# Supplementary material for: Retrospective analysis and time series forecasting with automated machine learning of ascariasis, enterobiasis and cystic echinococcosis in Romania
Source: PLoS Negl Trop Dis. 2021 Nov 1;15(11):e0009831. doi: 10.1371/journal.pntd.0009831 (PMC8584970; doi:10.1371/journal.pntd.0009831)
Supplement: S1 Table — Specific ICD-10 codes encoding either ascariasis, enterobiasis or cystic echinococcosis were selected according to the “ICD-10_AM diagnoses and procedures list” provided by the National School of Public Health, Management and Professional Development (NSPHMPDB) from Bucharest, Romania. (DOCX) [file pntd.0009831.s001.docx]

| **Parasitic NTD (pathogen)** | **ICD-10 code** |
| --- | --- |
| Ascariasis (***Ascaris lumbricoides*)** | B77.0; B77.8; B77.9 |
| Enterobiasis (***Enterobius vermicularis*)** | B80 |
| **Cystic echinococcosis (*Echinococcus granulosus*)** | B67.0; B67.1; B67.2; B67.3; B67.4 |

**S1 Table. Listing of ICD-10 codes selected for data extraction and preparation from the whole ICD-10 dataset of hospitalized patients in Romania during the period 2008-2018.** Specific ICD-10 codes encoding either ascariasis, enterobiasis or cystic echinococcosis were selected according to the “ICD-10_AM diagnoses and procedures list” provided by the National School of Public Health, Management and Professional Development (NSPHMPDB) from Bucharest, Romania.
