# Supplementary material for: Retrospective analysis and time series forecasting with automated machine learning of ascariasis, enterobiasis and cystic echinococcosis in Romania
Source: PLoS Negl Trop Dis. 2021 Nov 1;15(11):e0009831. doi: 10.1371/journal.pntd.0009831 (PMC8584970; doi:10.1371/journal.pntd.0009831)
Supplement: S2 Table — Regression equations for (A) ascariasis, (B) enterobiasis and (C) cystic echinococcosis. Reporting of regression equations for LSDV with a set of group dummy variables. The sets of group dummy variables were created to be able to compute regression equations that are specific to the NUTS 2 region. Each of the regions intercepts stands for the deviation of its group specific intercept from the intercept of the reference region. Example: To compute an approximation of the monthly ascariasis incidence rate in South East, the poverty rate can be inserted into the equation to obtain an approximation according to the fixed effects model used. In case of a poverty rate of 50%, a monthly ascariasis case rate of 0.539 (per 100,000) would be obtained. (DOCX) [file pntd.0009831.s002.docx]

| 1. **Regression equations for ascariasis** | |
| --- | --- |
| South East | Monthly ascariasis incidence rate = -5.111 + 0.113 * poverty rate |
| West | Monthly ascariasis incidence rate = -2.857 + 0.113 * poverty rate |
| Bucharest-Ilfov | Monthly ascariasis incidence rate = -3.043 + 0.113 * poverty rate |
| Center | Monthly ascariasis incidence rate = -1.37 + 0.113 * poverty rate |
| North East | Monthly ascariasis incidence rate = -2.657 + 0.113 * poverty rate |
| North West | Monthly ascariasis incidence rate = -0.427 + 0.113 * poverty rate |
| South Muntenia | Monthly ascariasis incidence rate = -2.9 + 0.113 * poverty rate |
| South West Oltenia | Monthly ascariasis incidence rate = -2.801 + 0.113 * poverty rate |
|  | |
| 1. **Regression equations for enterobiasis** | |
| South East | Monthly enterobiasis incidence rate = -1.417 + 0.043 * poverty rate |
| West | Monthly enterobiasis incidence rate = 0.85 + 0.043 * poverty rate |
| Bucharest-Ilfov | Monthly enterobiasis incidence rate = -0.4 + 0.043 * poverty rate |
| Center | Monthly enterobiasis incidence rate = -0.264 + 0.043 * poverty rate |
| North East | Monthly enterobiasis incidence rate = -0.732 + 0.043 * poverty rate |
| North West | Monthly enterobiasis incidence rate = 0.584 + 0.043 * poverty rate |
| South Muntenia | Monthly enterobiasis incidence rate = -0.736 + 0.043 * poverty rate |
| South West Oltenia | Monthly enterobiasis incidence rate = 1.175 + 0.043 * poverty rate |
|  | |
| 1. **Regression equations for cystic echinococcosis** | |
| South West Oltenia | Monthly cystic echinococcosis incidence rate = -1.345 + 0.038 * poverty rate |
| West | Monthly cystic echinococcosis incidence rate = -0.554 + 0.038 * poverty rate |
| Bucharest-Ilfov | Monthly cystic echinococcosis incidence rate = 3.305 + 0.038 * poverty rate |
| Center | Monthly cystic echinococcosis incidence rate = -0.599 + 0.038 * poverty rate |
| North East | Monthly cystic echinococcosis incidence rate = -1.325 + 0.038 * poverty rate |
| North West | Monthly cystic echinococcosis incidence rate = -0.512 + 0.038 * poverty rate |
| South Muntenia | Monthly cystic echinococcosis incidence rate = -1.31 + 0.038 * poverty rate |
| South East | Monthly cystic echinococcosis incidence rate = -1.345 + 0.038 * poverty rate |

**S2 Table. Regression equations for (A) ascariasis, (B) enterobiasis and (C) cystic echinococcosis.** Reporting of regression equations for LSDV with a set of group dummy variables. The sets of group dummy variables were created to be able to compute regression equations that are specific to the NUTS 2 region. Each of the regions intercepts stands for the deviation of its group specific intercept from the intercept of the reference region. Example: To compute an approximation of the monthly ascariasis incidence rate in South East, the poverty rate can be inserted into the equation to obtain an approximation according to the fixed effects model used. In case of a poverty rate of 50 %, a monthly ascariasis case rate of 0.539 (per 100,000) would be obtained.
