# Supplementary material for: Retrospective analysis and time series forecasting with automated machine learning of ascariasis, enterobiasis and cystic echinococcosis in Romania
Source: PLoS Negl Trop Dis. 2021 Nov 1;15(11):e0009831. doi: 10.1371/journal.pntd.0009831 (PMC8584970; doi:10.1371/journal.pntd.0009831)
Supplement: S3 Table — Regression tables for (A) ascariasis, (B) enterobiasis and (C) cystic echinococcosis. The regression tables were computed with Stata’s “xi” command, which converts categorical variables into dummy or indicator variables when fitting a model. The dependent variable, i.e. the monthly incidence rate of hospitalization of the respective disease, is measured as cases per 100,000. The reference region was chosen so that for the respective disease the coefficients of the categorical variables, the NUTS 2 regions, would be positive. A higher coefficient of a region would indicate a higher baseline case rate and vice versa. Therefore, the reference region accounts for the lowest baseline incidence rate. Next, a positive coefficient on the independent variable “poverty rate” indicates a positive correlation with the dependent variable and vice versa. Both R-squared values and the F-Test provide goodness-of-fit measures. (DOCX) [file pntd.0009831.s003.docx]

| Variables | Coef. | | St.Err. | t-value | | p-value | [95% Conf | | Interval] | | Sig | |
| --- | --- | --- | --- | --- | --- | --- | --- | --- | --- | --- | --- | --- |
| Poverty Rate | .113 | | .007 | 16.63 | | 0 | .1 | | .127 | | *** | |
| **Ref. (=Sud-Est)** | 0 | | . | . | | . | . | | . | |  | |
| West | 2.254 | | .15 | 15.06 | | 0 | 1.961 | | 2.548 | | *** | |
| Bucharest-Ilfov | 2.068 | | .175 | 11.82 | | 0 | 1.724 | | 2.411 | | *** | |
| Center | 3.741 | | .163 | 22.93 | | 0 | 3.421 | | 4.061 | | *** | |
| North East | 2.454 | | .123 | 19.97 | | 0 | 2.213 | | 2.695 | | *** | |
| North West | 4.684 | | .167 | 28.06 | | 0 | 4.357 | | 5.012 | | *** | |
| South Muntenia | 2.211 | | .127 | 17.43 | | 0 | 1.962 | | 2.46 | | *** | |
| S.-W. Oltenia | 2.31 | | .123 | 18.84 | | 0 | 2.07 | | 2.551 | | *** | |
| Constant | -5.111 | | .337 | -15.16 | | 0 | -5.772 | | -4.45 | | *** | |
|  | | | | | | | | | | | |  |
| R-squared | | 0.579 | | | Number of observations | | | 1056 | |  |  |  |
| F-test | | 179.969 | | | Prob > F | | | 0.000 | |  |  |  |
| **** p < 0.01, ** p < 0.05, * p < 0.1* | | | | | | | | | | | |  |
|  | | | | | | | | | | | |  |

1. **Regression table for ascariasis**
2. **Regression table for enterobiasis**

| Variable | Coef. | | St.Err. | t-value | | p-value | [95% Conf | | Interval] | | Sig |
| --- | --- | --- | --- | --- | --- | --- | --- | --- | --- | --- | --- |
| Poverty Rate | .043 | | .006 | 7.41 | | 0 | .032 | | .055 | | *** |
| **Ref. (=Sud-Est)** | 0 | | . | . | | . | . | | . | |  |
| West | 2.267 | | .128 | 17.71 | | 0 | 2.016 | | 2.518 | | *** |
| Bucharest-Ilfov | 1.017 | | .15 | 6.80 | | 0 | .724 | | 1.311 | | *** |
| Center | 1.153 | | .14 | 8.26 | | 0 | .879 | | 1.426 | | *** |
| North East | .685 | | .143 | 6.51 | | 0 | .479 | | .891 | | *** |
| North West | 2.001 | | .114 | 14.01 | | 0 | 1.721 | | 2.281 | | *** |
| South Muntenia | .681 | | .109 | 6.27 | | 0 | .468 | | .894 | | *** |
| S.-W. Oltenia | 2.592 | | .105 | 24.71 | | 0 | 2.386 | | 2.798 | | *** |
| Constant | -1.417 | | .288 | -4.91 | | 0 | -1.983 | | -.851 | | *** |
|  | | | | | | | | | | | |
| R-squared | | 0.487 | | | Number of observations | | | 1056 | |  |  |
| F-test | | 124.217 | | | Prob > F | | | 0.000 | |  |  |
| **** p < 0.01, ** p < 0.05, * p < 0.1* | | | | | | | | | | | |

1. **Regression table for cystic echinococcosis**

| Variable | Coef. | | St.Err. | t-value | | p-value | [95% Conf | | Interval] | | Sig |
| --- | --- | --- | --- | --- | --- | --- | --- | --- | --- | --- | --- |
| Poverty Rate | .038 | | .004 | 10.39 | | 0 | .031 | | .046 | | *** |
| **Ref. (=S.-V. Olt.)** | 0 | | . | . | | . | . | | . | |  |
| West | .791 | | .078 | 10.11 | | 0 | .637 | | .944 | | *** |
| Bucharest-Ilfov | 4.65 | | .091 | 50.99 | | 0 | 4.471 | | 4.829 | | *** |
| Center | .746 | | .085 | 8.77 | | 0 | .579 | | .913 | | *** |
| North East | .02 | | .067 | 0.30 | | 0.766 | -.112 | | .152 | | n.s. |
| North West | .833 | | .087 | 9.58 | | 0 | .663 | | 1.004 | | *** |
| South Muntenia | .035 | | .068 | 0.52 | | 0.603 | -.097 | | .168 | | n.s. |
| South East | .804 | | .066 | 12.11 | | 0 | .673 | | .934 | | *** |
| Constant | -1.345 | | .178 | -7.57 | | 0 | -1.694 | | -.997 | | *** |
|  | | | | | | | | | | | |
| R-squared | | 0.852 | | | Number of observations | | | 1056.000 | |  |  |
| F-test | | 751.148 | | | Prob > F | | | 0.000 | |  |  |
| **** p < 0.01, ** p < 0.05, * p < 0.1, n.s. = not signficant* | | | | | | | | | | | |

**S3 Table. Regression tables for (A) ascariasis, (B) enterobiasis and (C) cystic echinococcosis.** The regression tables were computed with Stata’s “xi” command, which converts categorical variables into dummy or indicator variables when fitting a model. The dependent variable, i.e. the monthly incidence rate of hospitalization of the respective disease, is measured as cases per 100,000. The reference region was chosen so that for the respective disease the coefficients of the categorical variables, the NUTS 2 regions, would be positive. A higher coefficient of a region would indicate a higher baseline case rate and vice versa. Therefore, the reference region accounts for the lowest baseline incidence rate. Next, a positive coefficient on the independent variable “poverty rate” indicates a positive correlation with the dependent variable and vice versa. Both R-squared values and the F-Test provide goodness-of-fit measures.
